# Supplementary material for: Benchmark performance of low-cost Sb2Se3 photocathodes for unassisted solar overall water splitting
Source: Nat Commun. 2020 Feb 13;11:861. doi: 10.1038/s41467-020-14704-3 (PMC7018841; doi:10.1038/s41467-020-14704-3)
Supplement: Supplementary file 1 — Supplementary Information [file 41467_2020_14704_MOESM1_ESM.pdf]

Supplementary Information

Benchmark performance of low-cost Sb_2Se_3 photocathodes for unassisted solar overall water splitting

By Yang et al.

Supplementary Figures

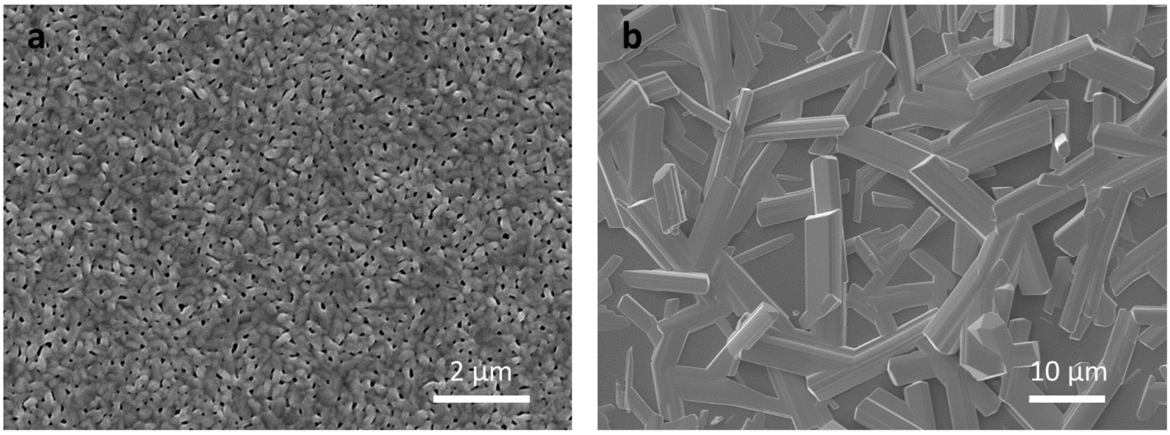

Supplementary Fig. 1 | Dependence of Sb_2Se_3 obtained via CSS on the source temperature. **a,** SEM image of compact Sb_2Se_3 film deposited at 340 °C. **b,** SEM image of 1D Sb_2Se_3 deposited at 460 °C.

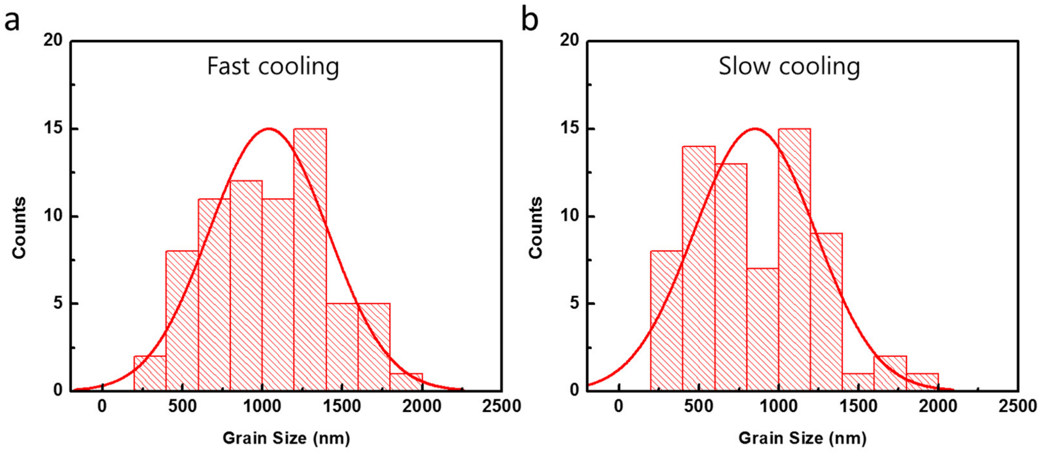

Supplementary Fig. 2 | Grain size distribution of Sb_2Se_3 thin films depending on the cooling rate. a, Fast cooling Sb_2Se_3 and **b**, slow cooling Sb_2Se_3. The fast cooling sample revealed a slightly larger average value of grain size (~1038 nm) compared with the one of the slow cooling (~850 nm), while both samples had similar standard deviation (~370 nm, Supplementary Fig. 2). In addition, the energy-dispersive X-ray spectroscopy (EDX) analysis showed that both fast and slow cooling Sb_2Se_3 films are slightly selenium poor (Se/Sb ~ 1.35) as similar with the previously reported CSS-Sb_2Se_3 thin films.

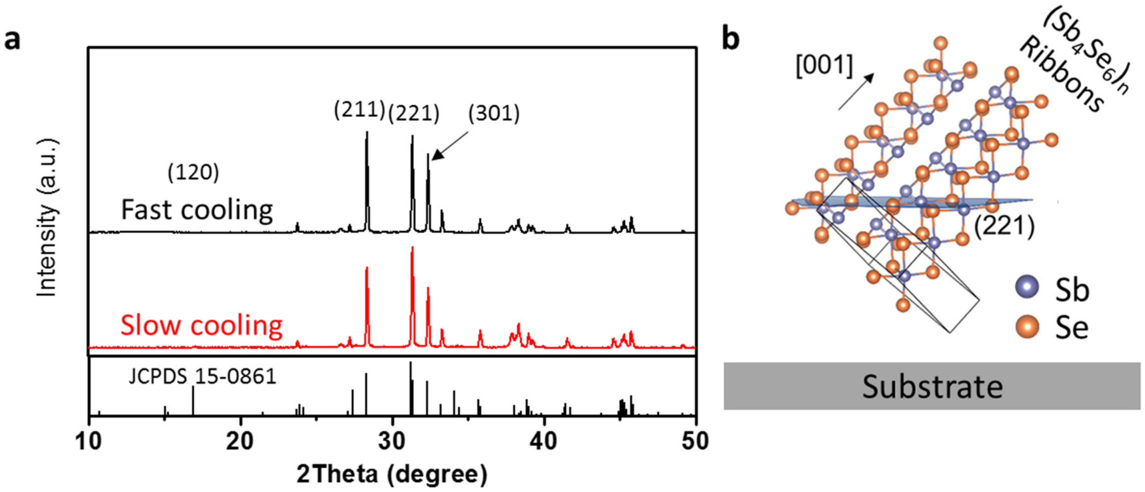

Supplementary Fig. 3 | Crystallographic orientations of Sb_2Se_3 thin films. a, XRD data for Sb_2Se_3 thin films. **b,** Schematic showing favourable orientation of $(Sb_4Se_6)_n$ ribbons. Source data used to generate this figure can be found in the Source Data file.

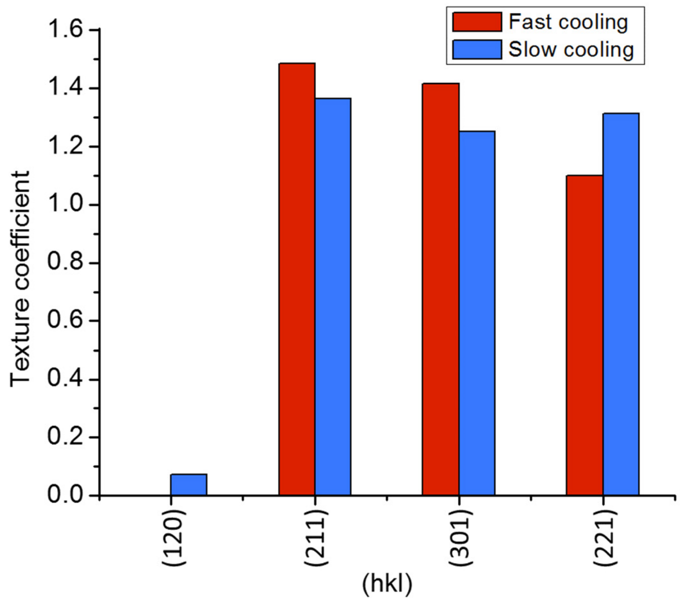

Supplementary Fig. 4 | The texture coefficients of selected diffraction peaks in different Sb_2Se_3 thin films. Further discussion is provided in Supplementary Note 1.

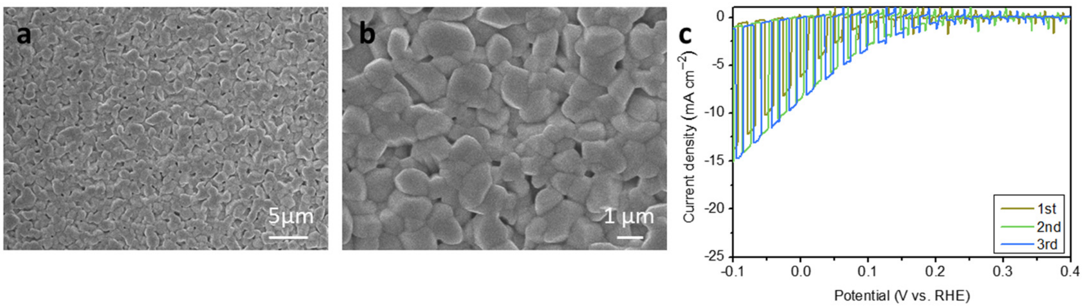

Supplementary Fig. 5 | Sb_2Se_3 photocathodes without the Au bottom contact layer. a-b, SEM images of fast-cooling Sb_2Se_3 on FTO substrate. The morphology of Sb_2Se_3 directly grown on the FTO substrate is nearly similar to that of Sb_2Se_3 grown on Au/FTO. **c**, J-V curves of $RuO_x/TiO_2/Sb_2Se_3$/FTO photocathodes in pH 1 H_2SO_4 electrolytes.

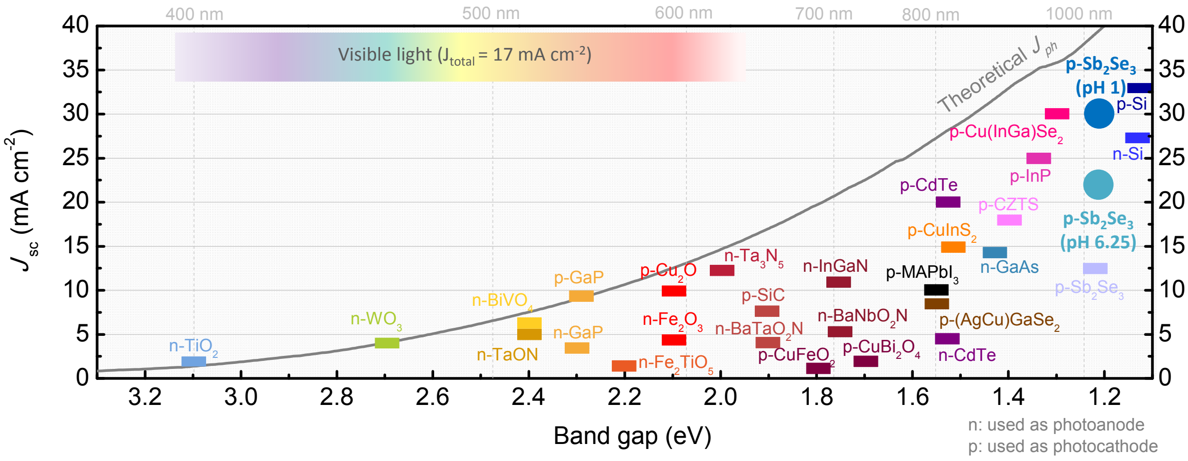

Supplementary Fig. 6 | Reported photocurrent density under short circuit condition (*Jsc*) of photoelectrodes for PEC water splitting under standard conditions: 1.0 sun, short circuit conditions of 1.23 V_{RHE} for photoanode, and 0 V_{RHE} for photocathode. Further discussion is provided in Supplementary Note 2. Reproduced by permission of The Royal Society of Chemistry[1].

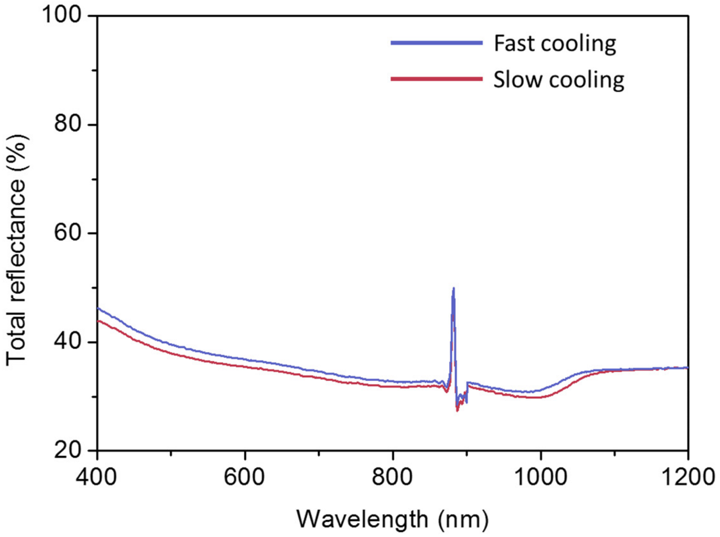

Supplementary Fig. 7 | Surface total reflectance (specular reflectance + diffusive reflectance) of Sb_2Se_3/Au/FTO.

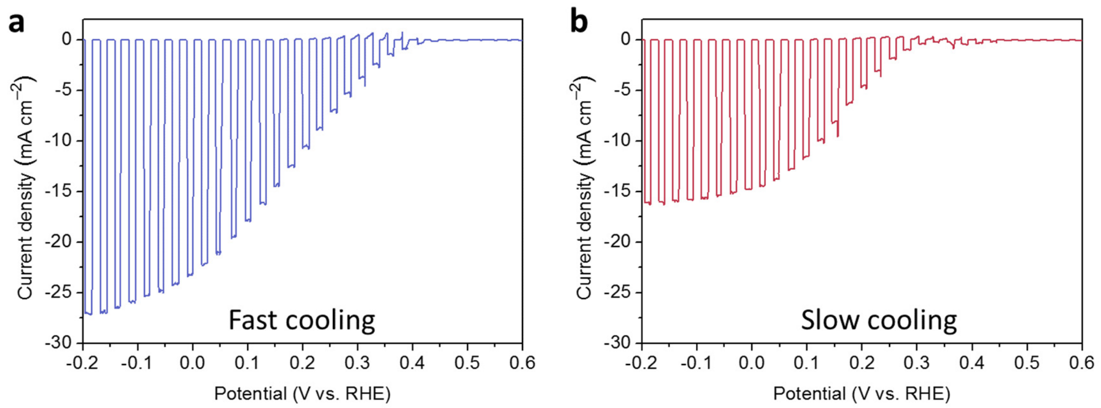

Supplementary Fig. 8 | J–V curves for Pt/TiO_2/Sb_2Se_3/Au/FTO photocathodes in aqueous H_2SO_4 electrolyte (pH ~ 1) under simulated 1 sun air mass 1.5 G chopped illumination at a scan speed of 5 mV s^{-1} in the cathodic direction.

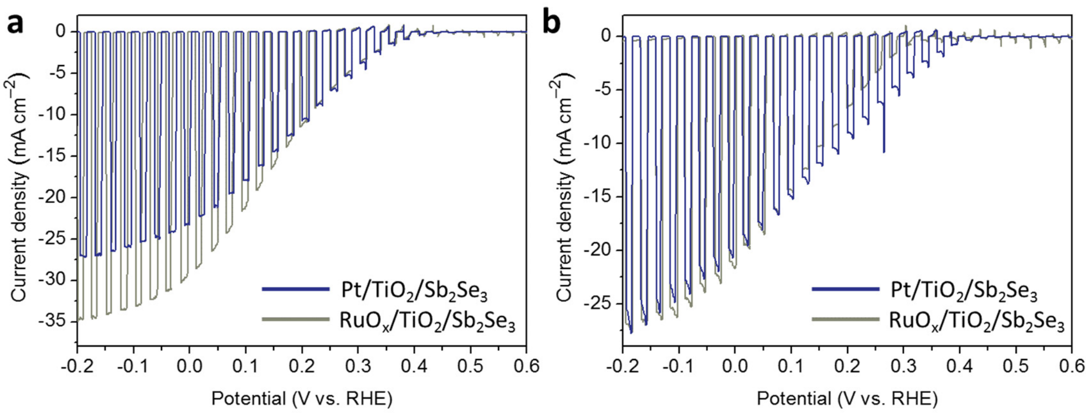

Supplementary Fig. 9 | J–V curves for Sb_2Se_3 photocathodes with two different co-catalysts **a,** in an acidic electrolyte (aqueous H_2SO_4, pH ~ 1) and **b,** in a neutral electrolyte (phosphate buffer, pH ~ 6.25). Further discussion is provided in Supplementary Note 3.

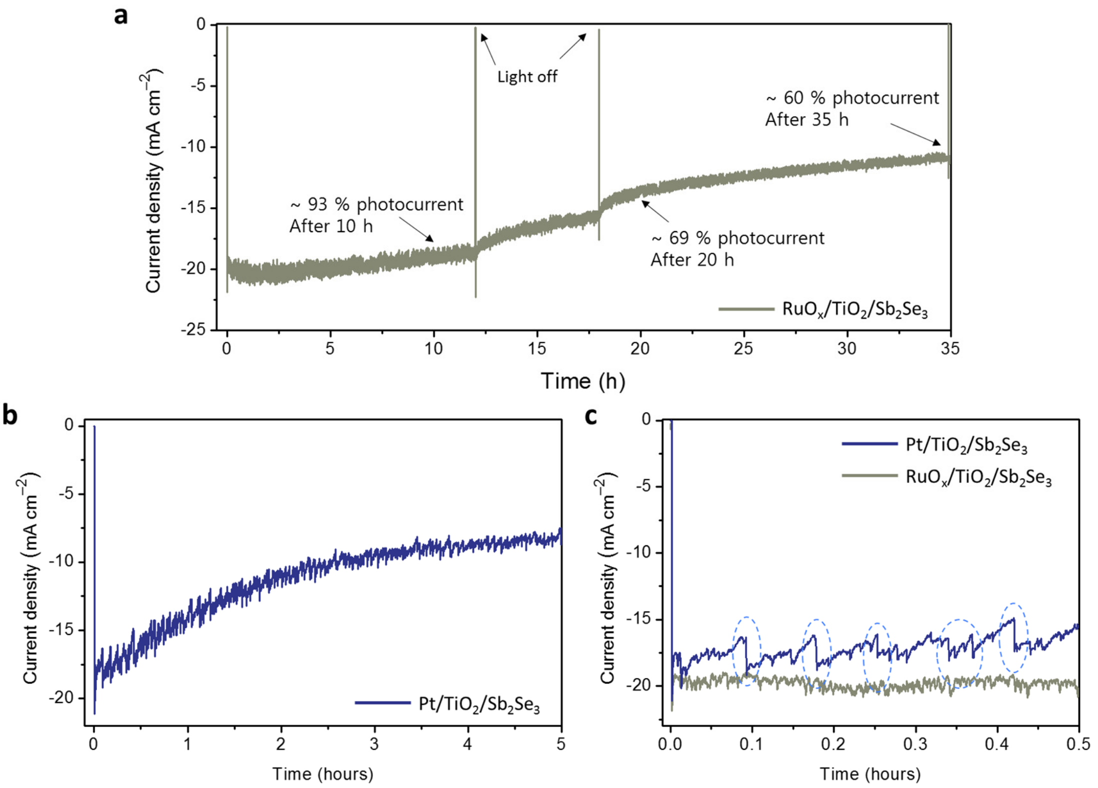

Supplementary Fig. 10 | Stability test results for Sb_2Se_3 photocathodes with two different co-catalysts in phosphate buffer (pH ~ 6.25). a, J–t curve for $RuO_x/TiO_2/Sb_2Se_3$ photocathode**. b,** J–t curve for $Pt/TiO_2/Se_3$ photocathode. **c,** Enlarged J–t curves for the Sb_2Se_3 photocathodes. Blue circles represent current fluctuation due to H_2 bubble detachment. Further discussion is provided in Supplementary Note 4.

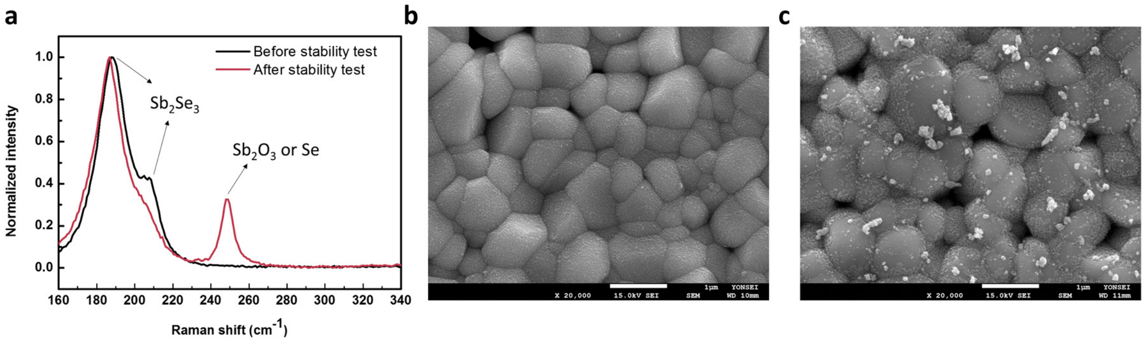

Supplementary Fig. 11 | Chemical composition and microstructures of Sb_2Se_3 before and after stability test. a, Raman spectra of Sb_2Se_3 photocathodes and SEM images of **b,** before and **c,** after stability test. Further discussion is provided in Supplementary Note 5.

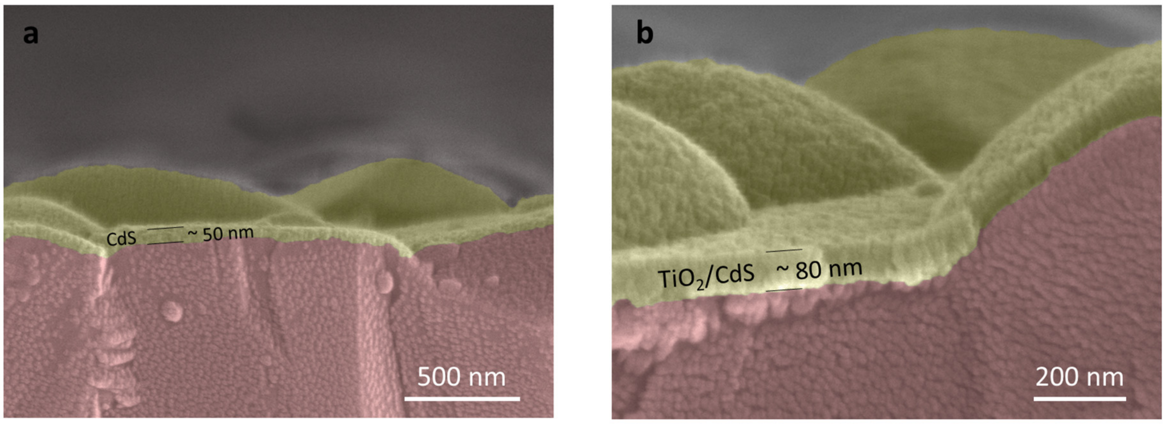

Supplementary Fig. 12 | Enlarged SEM images of overlayer-coated Sb_2Se_3. a, Image of CdS-coated Sb_2Se_3 showing thickness of the CdS layer (approximately 50 nm). **b,** Image of TiO_2/CdS coated on Sb_2Se_3 showing thickness of the TiO_2/CdS layer (approximately 80 nm). The thickness of the TiO_2 layer can be calculated as approximately ~30 nm, which is in agreement with the growth rate and deposition cycles (0.55 Å and 600 cycles).

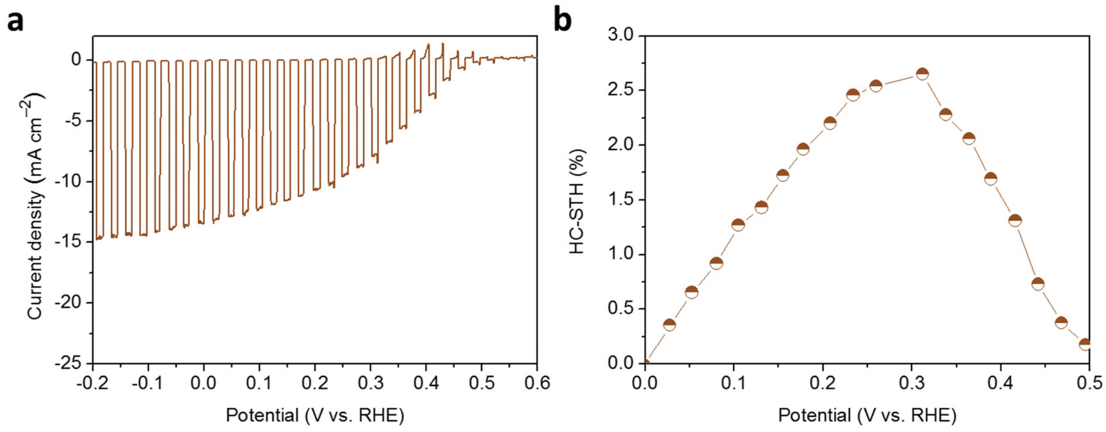

Supplementary Fig. 13 | PEC performance of Pt/TiO_2/CdS/Sb_2Se_3/Au/FTO photocathode in pH 1 electrolyte with thick CdS layer. a, J–V curve and **b,** corresponding HC-STH efficiency. The CdS layer was deposited by CBD for 7 min (the optimum deposition time was 5 min).

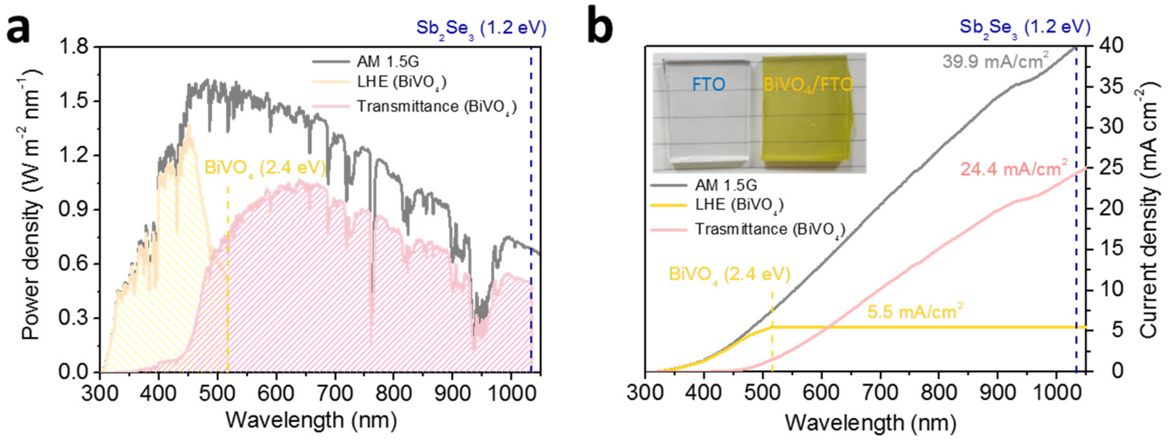

Supplementary Fig. 14 | Optical properties of $BiVO_4$ film. a, AM 1.5 G spectrum and corresponding region of light harvesting efficiency and transmittance and **b,** calculated current density for $BiVO_4$.

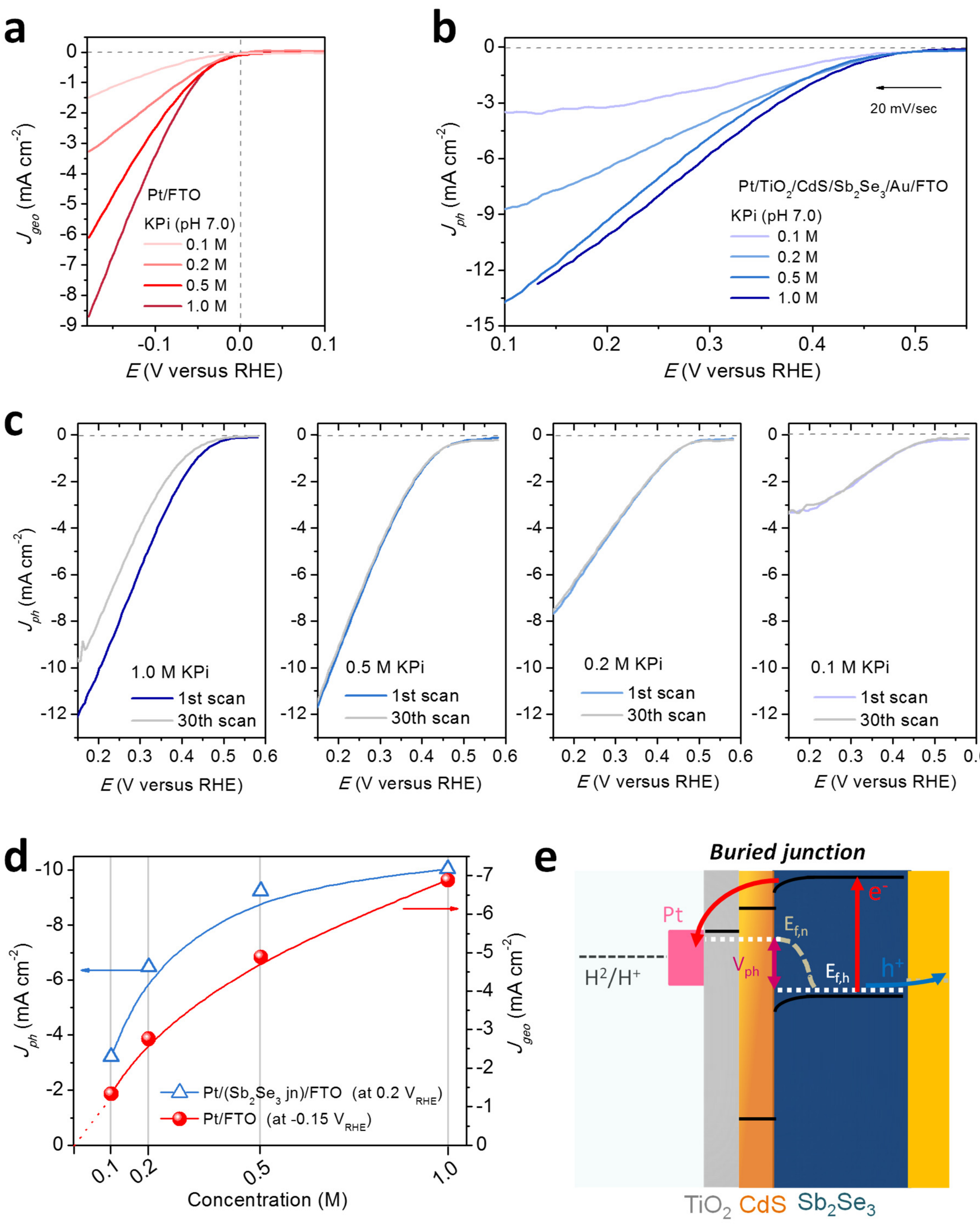

Supplementary Fig. 15 | Scans for optimum electrolyte concentration for the photocathode. J–V curves for different concentrations of phosphate buffer. **a,** Pt/FTO electrode and **b,** Pt/junctioned Sb_2Se_3 photocathode. **c,** Repeated scans for Pt/junctioned Sb_2Se_3 for verifying short term (< 600 s) stability. **d,** Current density profile for the (photo)electrodes for different concentrations. **e.** Proposed charge transfer mechanism of Pt/junctioned Sb_2Se_3.

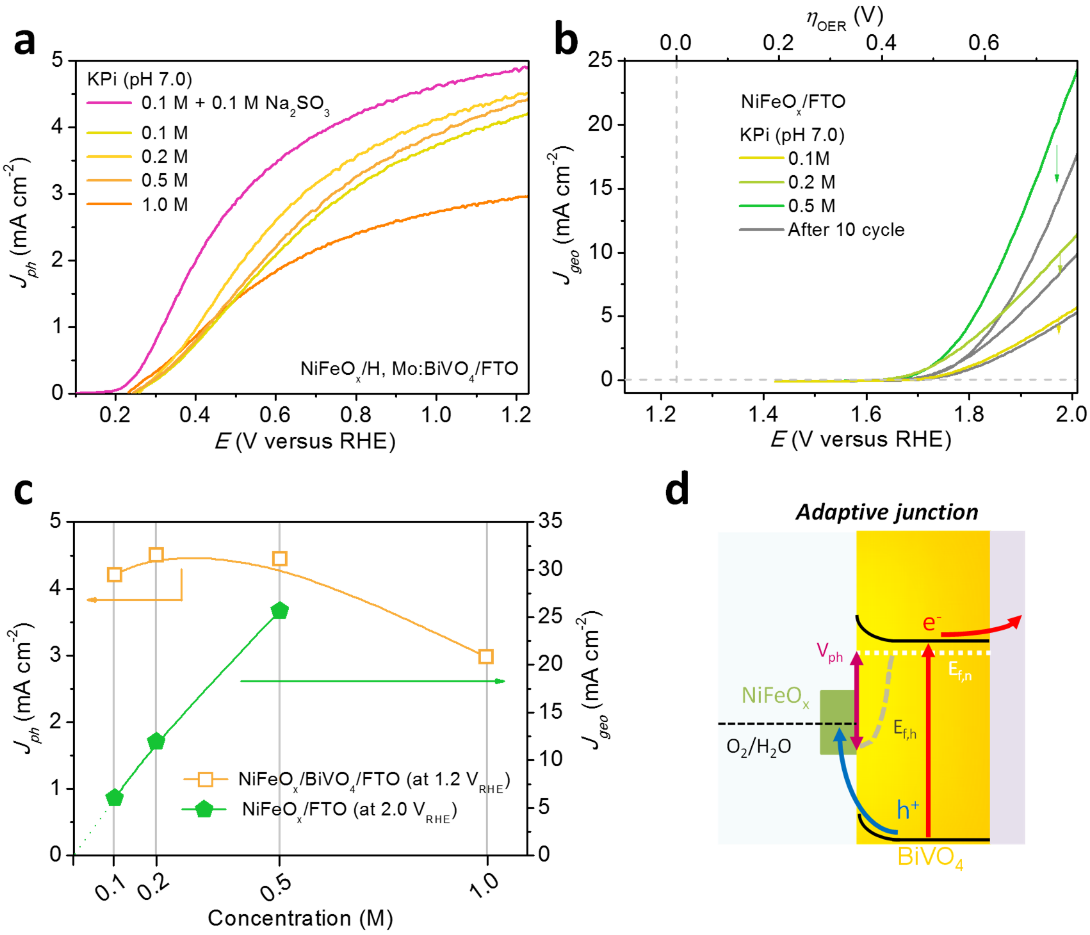

Supplementary Fig. 16 | Scans for optimum electrolyte concentration for the photoanode. J–V curves for different concentrations of phosphate buffer. **a,** $NiFeO_x/BiVO_4$ photoanode and **b,** $NiFeO_x$/FTO electrode. **c,** Current density profile for the (photo)electrodes with different concentrations. **d,** Proposed charge transfer mechanism of $NiFeO_x/BiVO_4$.

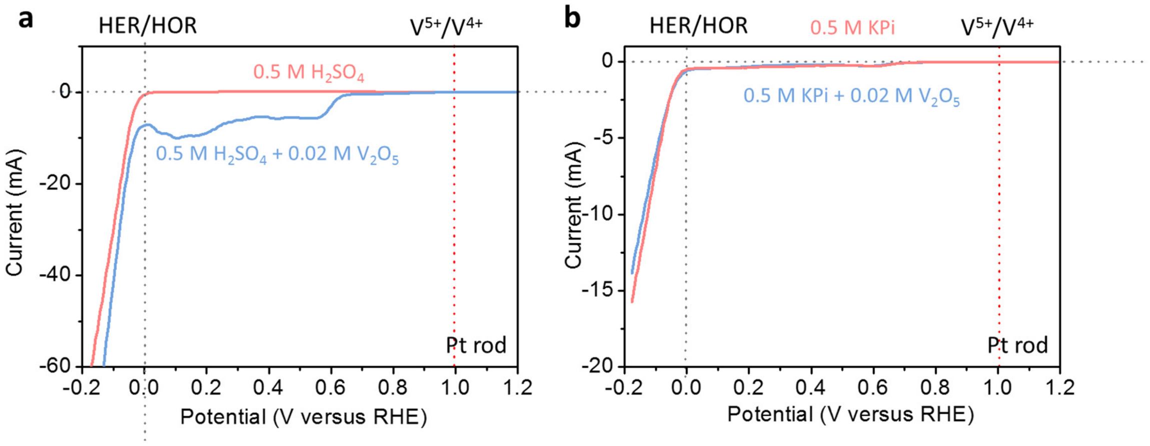

Supplementary Fig. 17 | Linear sweep voltammogram of Pt rod with/without V^{5+} in (a) a strongly acidic electrolyte and (b) a neutral electrolyte.

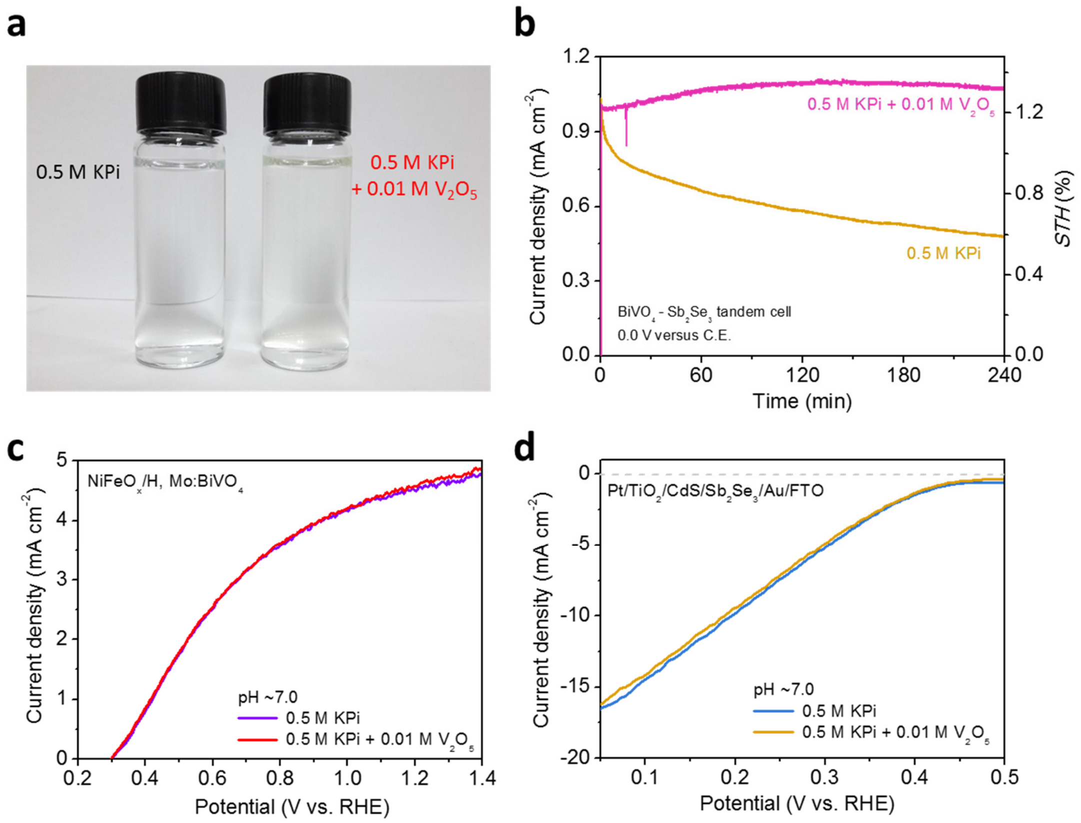

Supplementary Fig. 18 | a, Photograph of vanadium oxide-dissolved phosphate buffer. A slightly yellow hue was observed. **b,** Stability of two $BiVO_4$–Sb_2Se_3 tandem cells in different electrolytes at a constant potential (0.0 V against the counter electrode). **c-d,** J–V curves for each photoelectrode without or with dissolved vanadium oxide. Further discussion is provided in Supplementary Note 6.

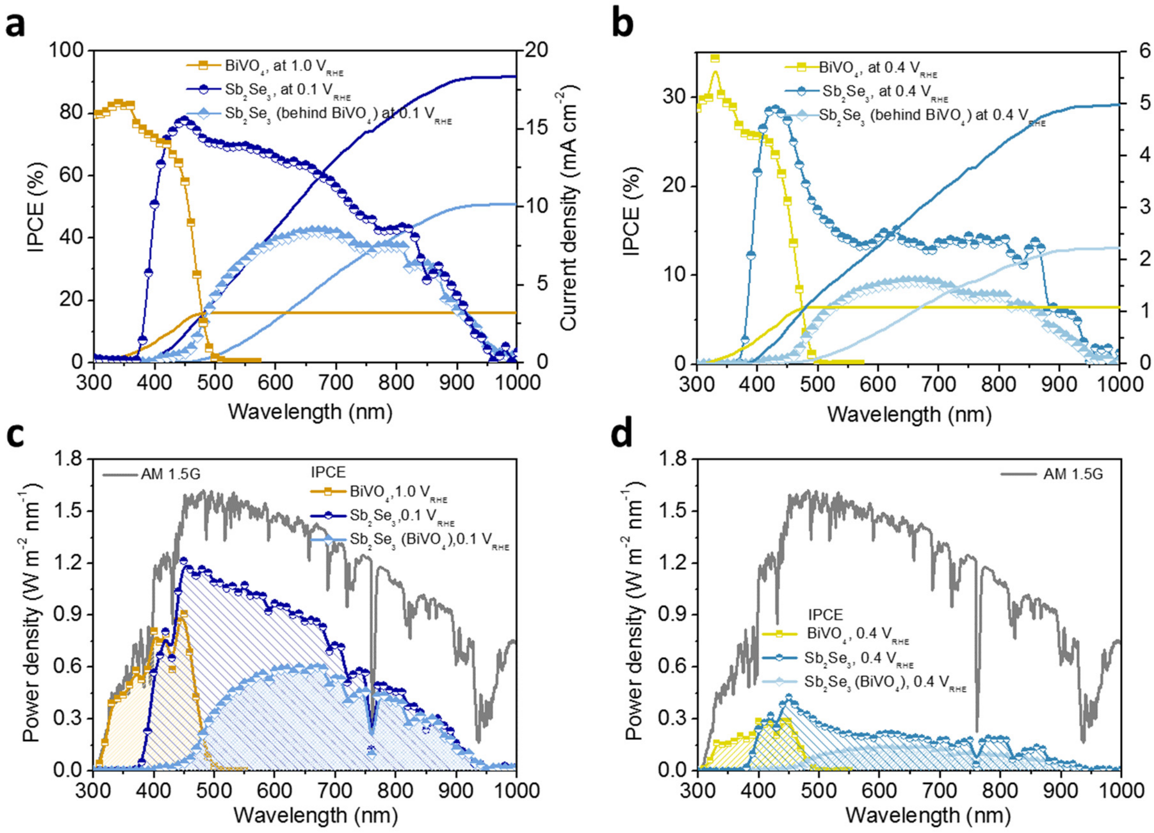

Supplementary Fig. 19 | IPCE and calculated photocurrent density at a, 1.0 V_{RHE} for photoanode and 0.1 V_{RHE} for photocathode and **b,** 0.4 V_{RHE} for both photoelectrodes. **c-d,** Calculated power density curves for each photoelectrode against AM 1.5G spectrum.

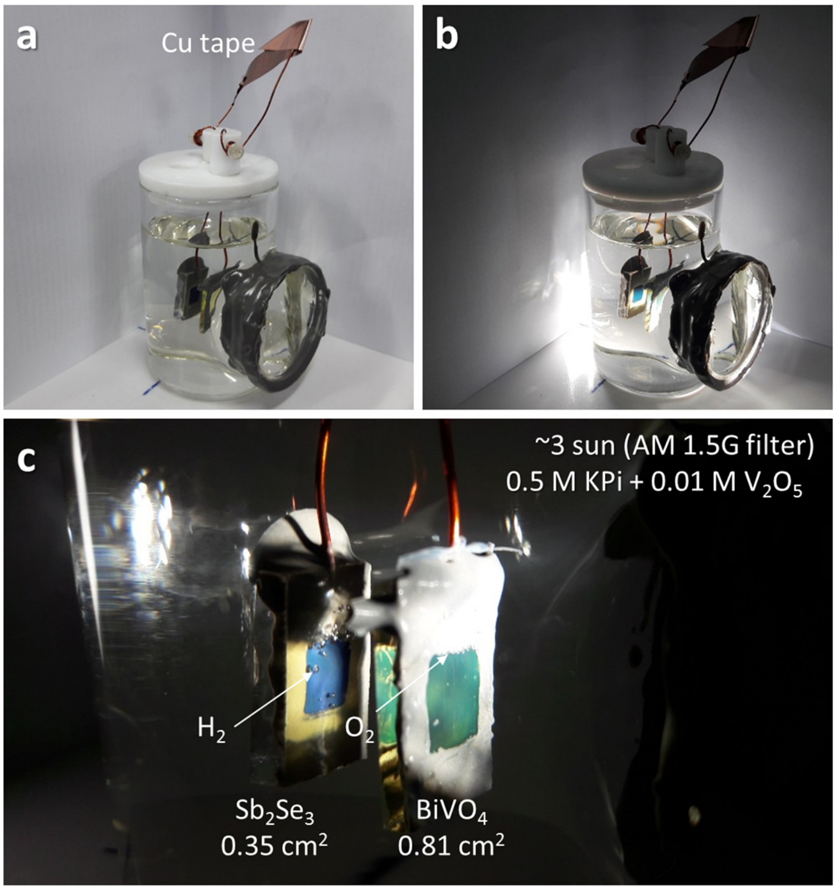

Supplementary Fig. 20 | Visual demonstration of $BiVO_4$-Sb_2Se_3 tandem cell. Electrolyte: 0.5M KPi + 0.01 M V_2O_5 (pH 7.0). Illumination source was set at ~3 sun to accelerate the demonstration. Supplementary Video 1 offers further elucidation.

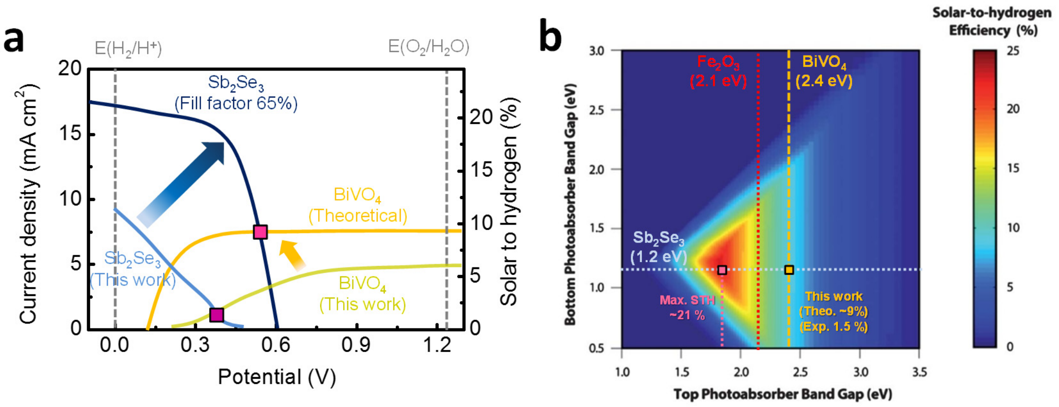

Supplementary Fig. 21 | a, Experimentally obtained and hypothetical J–V curves for $BiVO_4$ and Sb_2Se_3 to predict operating point. **b,** Assorted light absorber position on theoretically obtained plot for the D4 scheme. Reproduced from Supplementary Ref. 2 with permission from The Royal Society of Chemistry.

Supplementary Tables

Table 1. Performances of previously reported photocathode–photoanode D4 cells in overall water splitting (2012–2019).

*List	Photocathode	Photoanode	**Electrolyte (pH)	***Efficiency	Ref
**(1)**	**$Pt/TiO_2/CdS/Sb_2Se_3/Au/FTO$**	**$NiFeO_x/H$, $Mo:BiVO_4$**	**0.5 M KPi + 0.01 M V_2O_5 pH 7.0**	**1.5%, stable for 10 h**	**This work**
(2)	TiNi/p-Si	$TiCo/BiVO_4$	0.1 M KPi (pH 7.0)	Tandem scheme HC-STH: 0.05%	3
(3)	Pt/p-Si	$Co\text{-}Pi/Mo:BiVO_4$	0.5 M KPi (pH 7.0)	Tandem scheme STH: 0.57%	4
(4)	$Pt/TiO_2/a\text{-}Si$	$NiFeO_x/Fe_2O_3$	0.5 M KPi (pH 11.8)	Tandem scheme STH: 0.91%, stable for 10 h	5
(5)	$NiMo+SiO_2$(partially coated)/n/p-Si nanowire	$NiOOH/FeOOH/Mo:BiVO_4$	0.5 M KCi (pH 10) (anode)/ 0.1 M H_2SO_4 (pH 1) (cathode)	Tandem scheme STH: 2.1%, stable for 2 h	6
(6)	$Pt/TiO_2/Zn:InP$	$Co\text{-}Pi/BiVO_4$	0.1 M KPi (pH 7)	Parallel scheme STH: 0.5%	7
(7)	$Pt/In_2S_3/CdS/Cu_2ZnSnS_4$ Mo/SLG	$NiOOH/BiVO_4$	0.2 M NaPi (pH 6.5)	Parallel scheme HC-STH: 0.28%	8
(8)	$Pt/Mo/Ti/CdS/In_2S_3/(ZnSe)_{0.85}(CuIn_{0.7}Ga_{0.3}Se_2)_{0.15}$/Mo/SLG/Ti foil	$NiFeO_x/BiVO_4$	1.0 M KBi (pH 9.2)	Parallel scheme STH: 1.0%	9
(9)	$Pt/Mo/Ti/(ZnSe)_{0.85}(CIGS)_{0.15}$/Mo/SLG	$NiFeO_x/BiVO_4$	0.5 M KBi (pH 9.5)	Parallel scheme STH: 0.91%	10
(10)	$Pt/ZnS/CdS/(ZnSe)_{0.85}(CuIn_{0.7}Ga_{0.3}Se_2)_{0.15}$/Mo/SLG	$NiFeO_x/BiVO_4$	0.5 M KBi (pH 9.5)	Parallel scheme STH: 0.6%	11
(11)	$Pt/CdS/CuGa_3Se_5/(Ag,Cu)GaSe_2$/Mo/SLG	$NiOOH/FeOOH/Mo:BiVO_4$	0.1 M KPi (pH 7)	Tandem scheme STH: 0.67%, stable for 2 h	12
(12)	$Pt/TiO_2/CdS/(CuGa_{1-y}In_y)_{1-x}Zn_{2x}S_2/Au$	$CoFeO_x/BiVO_4$	0.25 M KPi (pH 7)	Parallel scheme STH: 1.1%	13
(13)	$Pt/HfO_2/CdS/Cu_2ZnSnS_4$ Mo/SLG	$NiFeO_x/BiVO_4$	0.2 M NaPi (pH 6.5)	STH: 1.046%, stable for 10 h	14
(14)	$Pt/TiO_2/Al_2O_3/CdS/CIGS$/Mo/SLG	$NiOOH/FeOOH/BiVO_4$	0.5 M KBi, pH 9.2	STH: 1.01%, stable for >0.5 h	15
(15)	$Pt/TiO_2/CdS/CuIn_{0.5}Ga_{0.5}Se_2$/Mo/SLG	$NiFeO_x/BiVO_4$	0.5 M KBi, pH 9.3	STH: 3.7%, stable for >0.5 h	16
(16)	$Pt/In_2S_3/CdS/(ZnSe)_{0.85}(CuIn_{0.7}Ga_{0.3}Se_2)_{0.15}$/Mo/SLG	Anion exchange isomer/$NiFeO_x$/$BiVO_4$	0.5 M KBi+ cation chelating agent pH 9.3	STH: 1.0%, stable for 50 h (60% retained)	17
(17)	$RuO_2/TiO_2/AZO/Cu_2O/Au/FTO$	$Co\text{-}Pi/W:BiVO_4$	0.5 M KPi (pH 7.0)	Tandem scheme STH: 1.0% (retained less than 5 min)	18
(18)	$RuO_2/TiO_2/Ga_2O_3/Cu_2O/Au/FTO$	$NiFeO_x$/reduced $Mo:BiVO_4$	0.5 M KBi (pH 9.0)	STH: 3.0%, stable for 12 h (90% retained)	19
(19)	$Pt/CuBi_2O_4/FTO$	$Co\text{-}Pi/Mo:BiVO_4$	0.1 M KPi (pH 7.0)	STH: 0.15% Unstable	20

(20)	Pt/Ag/PEIE/PCBM/(CsFA MA)PbI_3/NiO/FTO	TiCo/$BiVO_4$	0.5 M KBi+ K_2SO_4 pH 8.5	STH: 0.59%, stable for 18 h	21

*Reports are cited chronologically, but not according to the actual publication date.
**KPi: potassium biphosphate, NaPi: sodium biphosphate, KBi: Potassium borate
***Light source for all experiments was AM 1.5G, 100 mW/cm^2 (1 sun) unless noted otherwise. Material was denoted as stable if constant potential operation was demonstrated without significant drop in efficiency (< 25%).

Supplementary Note

Supplementary Note 1

Supplementary Fig. 3a shows the XRD data obtained for both fast-cooling and slow-cooling samples: it reveals the preferred (hk1) orientations (i.e. strong (211), (221), and (301) peaks with a negligible (120) peak (*pbnm space group*)). As found in previous studies on Sb_2Se_3 thin-film solar cells, (hk1) orientations, representing $(Sb_4Se_6)_n$ nanoribbons oriented perpendicular or inclined relative to the substrate (Supplementary Fig. 3b, for example), are advantageous for a superior performance owing to efficient carrier transport along the [001] direction[22,23]. To quantify the relative intensities of each plane revealed in XRD data to a standard Sb_2Se_3 powder (JCPDS 15-8601), we have calculated the texture coefficient T_c, which is defined as:

$$T_c(hkl) = n \frac{I(hkl)/I_o(hkl)}{\sum_1^n I(hkl)/I_o(hkl)}$$

where *I(hkl)* is the measured relative intensity of the peak corresponding to the *hkl* diffraction, $I_o(hkl)$ is the relative intensity from a standard powder sample (JCPDS 15-0861), and *n* is the total number of diffraction peaks used in the evaluation. A large T_c value for a specific diffraction peak indicates preferred orientation along this direction. In the present case, we chose four diffraction peaks (n = 4) corresponding to 2θ values of 120, 211, 221, and 301. Supplementary Fig. 4 clearly shows that T_c (120) of both fast and slow cooling samples is nearly zero while the other values are higher than 1, indicating both samples have (hk1) preferred orientation. Although both samples have a similar preferred orientation, it is also obvious that the fast cooling sample revealed higher T_c values of (211) and (301) planes and lower T_c value of (221) plane, implying possible rearrangement of the ribbons.

Supplementary Note 2

Supplementary Fig. 6 shows the previously reported photocurrent densities of photoelectrodes for PEC water splitting under short circuit conditions (i.e. 1.23 V_{RHE} for photoanode and 0 V_{RHE} for photocathode) plotted with the theoretical maximum photocurrent density as a function of the band gap of the semiconductor materials. Although the photocurrent density of some large-E_g semiconductors such as TiO_2, WO_3, and GaP has reached the theoretical maximum, it has remained below 10 mA cm^{-2} in such cases due to their large E_g. One of the highest photocurrent density values was achieved by a p-type Si based photocathode (~35.5 mA cm^{-2} at 0 V_{RHE})[6]. However, considering the small band gap of Si (~1.1 eV) and the established history of the Si-based industry, the high photocurrent density obtained by the Si photocathode does not represent a remarkable result. Moreover, due to the low α of crystalline Si, complex microwire structures with sufficient thickness (p-Si wafer of 525 μm and Si microwires of 40 μm) and high-purity single-crystals are indispensable for an efficient Si photocathode. In addition to the Si photocathodes, a high photocurrent of ~30 mA cm^{-2} at 0 V_{RHE} was exhibited by the thin (1–1.5 μm) and polycrystalline p-Cu(In,Ga)Se_2 photocathode[24]. Despite the high photocurrent density, however, the use of expensive In and Ga could be a major bottleneck for the fabrication of large-area specimens.

It is also worth emphasizing that the most important building block in a PEC water splitting device in terms of cost-effectiveness is the light-absorbing semiconductors. For example, the cost portion of electrocatalysts in the c-Si based PV-EC system is just approximately 1 % (whether the electrocatalyst is Ir-Ru or NiFe-NiMo) for overall cost of hydrogen production[25]. Additionally, in the high STH systems based on III–V semiconductors, the cost of InGaP/GaAs ($175) per unit solar collection area is much greater than other parts such as catalysts (Pt and IrO_x, $8) and membranes (127 mm-thick Nafion, $5)[26]. Moreover, the community is recognizing

that noble metals are easy to recycle, as well as price of hydrogen expected from non-noble metal electrocatalysts is not competitive to the one with noble metal ones[27]. Thus, despite the use of relatively expensive catalysts and a hole selective contact layer, the high performance of our device demonstrates the feasibility of cost-effective Sb_2Se_3 based photoelectrodes for PEC water splitting.

Supplementary Note 3

The primary crucial issue is electrolyte compatibility because most of the efficient photoanodes reveal high performance in a near-neutral electrolyte, while our Sb_2Se_3 photocathodes were tested in harsh acidic electrolytes. We measured the PEC performance of our fast-cooling Sb_2Se_3 photocathode with two different co-catalysts, Pt and RuO_x, in both acidic (Supplementary Fig. 9a) and neutral (Supplementary Fig. 9b) electrolytes. In the acidic electrolyte, both RuO_x and Pt co-catalysts exhibited the same onset potential whereas a lower photocurrent density was observed in the Pt-coated sample. The lower photocurrent in the Pt co-catalyst sample was attributed to less light absorption and reflection by Pt nanoparticles, which is often quoted as a major issue associated with the use of Pt co-catalysts in the PEC system[28]. A more delicate strategy for depositing Pt co-catalyst uniformly, such as the two-step platinisation method[15], could boost the performance of our $Pt/TiO_2/Sb_2Se_3$ photocathodes.

Supplementary Note 4

Another interesting difference between RuO_x and Pt was the stability. The $RuO_x/TiO_2/Sb_2Se_3$ sample retained approximately 60% of initial photocurrent density after 35 h in the neutral electrolytes, which is the best stability of Sb_2Se_3 photocathodes reported so far (Supplementary Fig. 10a). The photocurrent density of $Pt/TiO_2/Sb_2Se_3$ decreased more rapidly (42% photocurrent after 5 h), probably due to the larger bubbles at Pt surfaces, as evidenced by severe fluctuations in the enlarged photocurrent curves (Supplementary Fig. 10b–c). It should be noted that the detachment of Pt particles due to the releasing of large bubbles is one of the well-known degradation mechanisms in the Pt-decorated photocathodes for water splitting[29].

Supplementary Note 5

We measured Raman spectroscopy to investigate the chemical composition variation after the reliability test. Before the stability test, the Raman spectra of the $RuO_x/TiO_2/Sb_2Se_3$/Au/FTO photocathode showed one distinct peak at ≈ 190 cm^{-1} along with a shoulder peak at ≈ 208 cm^{-1}, both of which are attributed to the vibration modes in Sb_2Se_3 phase (Supplementary Fig. 11a). After the stability test, an additional peak located at ≈ 250 cm^{-1} appeared. The additional peak indicates the formation of by-products such as Sb_2O_3 (≈ 254 cm^{-1}) and/or several Se phases (e.g., Se_8 rings at ≈ 253 cm^{-1}, Se_6 rings at ≈ 247 cm^{-1}, and amorphous Se at ≈ 250 cm^{-1}) as a result from the decomposition of Sb_2Se_3. In addition, there was also morphological destruction after the stability test (Supplementary Fig. 11b-c). According to our previous study on the stability of Sb_2Se_3 photocathodes[30], the morphological destruction of Sb_2Se_3 photocathode is caused by the photo-reduction of TiO_2 accompanied by the degradation of Sb_2Se_3. Despite the high photocurrent density and high stability of the RuO_x sample, it resulted in a smaller onset potential compared with that owing to the Pt sample due to the low catalytic activity of RuO_x in a neutral electrolyte (Supplementary Fig. 9b).

Supplementary Note 6

We tested various concentrations of phosphate buffer (0.1–1.0 M) to investigate its effect on the photocathode (Supplementary Fig. 15) and photoanode (Supplementary Fig. 16). For the Sb_2Se_3 photocathode, because the surface charge transfer kinetics is governed by Pt and photovoltage is achieved by the buried junction (i.e. TiO_2/CdS/Sb_2Se_3), a more conductive (higher concentration) electrolyte showed better performance[16]. On comparing Pt/TiO_2/CdS/Sb_2Se_3/Au/FTO and Pt/FTO, they exhibited an almost linear dependence of photocurrent density on the concentration of phosphate buffer (Supplementary Fig. 15d). Onset potential of the two (photo)electrodes (i.e. Pt/FTO and Pt/TiO_2/CdS/Sb_2Se_3/Au/FTO) showed negligible change while the current density varied greatly; from the concentration of 0.1 M to 0.5 M, the Sb_2Se_3 photocathode revealed a three-fold increment while the Pt electrocatalyst showed a five-fold increase in the current density. However, at a high concentration of 1.0 M, the stability of the photocathode degraded presumably due to the accelerated Pt detachment in the higher photocurrent condition (Supplementary Fig. 15c), so that 0.5 M could be a judicious choice for the photocathode considering both the photocurrent and the stability. For the photoanode, the completely opposite tendency was observed: the photocurrent of $NiFeO_x$/FTO (as an electrocatalyst) showed a linear dependence on the concentration, but that of $NiFeO_x$/$BiVO_4$/FTO (as a photoanode) revealed almost no dependence on the electrolyte concentration (Supplementary Fig. 16). This is due to the working mechanism of $NiFeO_x$/$BiVO_4$, which formed an adaptive junction with water-permeable $NiFeO_x$ showing two different effects of passivation and alternative active sites for improving the surface hole transfer[31,32]. Therefore, for the concentrations we tested (0.1–1.0 M), no photocurrent enhancement occurred for a higher concentration for the $NiFeO_x$/$BiVO_4$ photoanodes, so that the use of higher concentration of electrolyte had no beneficial effect. In fact, a higher electrolyte concentration could assist the corrosion of the electrocatalyst and photoanode (dissolution of $BiVO_4$ in phosphate buffer is spontaneous and accelerated by surface

photo oxidation[33]). Therefore, it would be desirable to use a 0.5 M phosphate buffer as an operating electrolyte that provides moderately benign conditions for both photoelectrodes. However, owing to the low stability of $BiVO_4$ in phosphate, fast degradation of performance was observed for the tandem cell, and we addressed the stability issue by adding vanadium cation (V^{5+}) as done by Choi group[34]. It should be noted that theoretically the V^{5+} can be reduced prior to proton, possibly affecting the performance of our Sb_2Se_3 photocathode-based tandem devices for water splitting. As shown in Supplementary Fig. 17a, there are distinctive peaks in the LSV scans for a Pt electrode upon addition of V^{5+} into strongly acidic electrolyte, indicative of a significant reduction of V^{5+}. In contrast, there is no noticeable difference between with/without V^{5+} electrolyte when measured in a neutral electrolyte (0.5 M KPi, Supplementary Fig. 17b). These results imply that the reactivity of V^{5+}, which is relatively stronger in an acidic electrolyte, significantly decreases in a neutral electrolyte. As we measured our Sb_2Se_3-based tandem device in a neutral electrolyte (0.5 M KPi), there is no significant change of both the Sb_2Se_3 photocathode and the $BiVO_4$ photoanode upon adding V^{5+} into our electrolyte as shown in Supplementary Fig. 18. It is also noteworthy that the slight difference observed in the photocathode case (Supplementary Fig. 18d), possibly due to parasitic light absorption by yellow V^{5+} ions, does not affect the performance of our tandem device as the operation potential of the tandem device is around 0.4 V_{RHE}. Accordingly, in any cases, it is reasonable to conclude that addition of V^{5+} does not interfere with the hydrogen production by our Sb_2Se_3-based tandem device.

Supplementary References

1 Kim, J. H., Hansora, D., Sharma, P., Jang, J.-W. & Lee, J. S. Toward practical solar hydrogen production – an artificial photosynthetic leaf-to-farm challenge. *Chem. Soc. Rev.* **48**, 1908-1971 (2019).

2 Pinaud, B. A. *et al.* Technical and economic feasibility of centralized facilities for solar hydrogen production via photocatalysis and photoelectrochemistry. *Energy Environ. Sci.* **6**, 1983-2002 (2013).

3 Lai, Y.-H., Palm, D. W. & Reisner, E. Multifunctional coatings from scalable single source precursor chemistry in tandem photoelectrochemical water splitting. *Adv. Energy Mater.* **5**, 1501668 (2015).

4 Xu, P. *et al.* Photoelectrochemical cell for unassisted overall solar water splitting using a $BiVO_4$ photoanode and Si nanoarray photocathode. *RSC Adv.* **6**, 9905-9910 (2016).

5 Jang, J.-W. *et al.* Enabling unassisted solar water splitting by iron oxide and silicon. *Nat. Commun.* **6**, 7447 (2015).

6 Vijselaar, W. *et al.* Spatial decoupling of light absorption and catalytic activity of Ni–Mo-loaded high-aspect-ratio silicon microwire photocathodes. *Nat. Energy* **3**, 185-192 (2018).

7 Kornienko, N. *et al.* Growth and photoelectrochemical energy conversion of wurtzite indium phosphide nanowire arrays. *ACS Nano* **10**, 5525-5535 (2016).

8 Jiang, F. *et al.* $Pt/In_2S_3/CdS/Cu_2ZnSnS_4$ thin film as an efficient and stable photocathode for water reduction under sunlight radiation. *J. Am. Chem. Soc.* **137**, 13691-13697 (2015).

9 Higashi, T. *et al.* Overall water splitting by photoelectrochemical cells consisting of $(ZnSe)_{0.85}(CuIn_{0.7}Ga_{0.3}Se_2)_{0.15}$ photocathodes and $BiVO_4$ photoanodes. *Chem. Commun.* **53**, 11674-11677 (2017).

10 Kaneko, H. *et al.* A novel photocathode material for sunlight-driven overall water splitting: solid solution of ZnSe and $Cu(In,Ga)Se_2$. *Adv. Funct. Mater.* **26**, 4570-4577 (2016).

11 Goto, Y. *et al.* A particulate $(ZnSe)_{0.85}(CuIn_{0.7}Ga_{0.3}Se_2)_{0.15}$ photocathode modified with CdS and ZnS for sunlight-driven overall water splitting. *J. Mater. Chem. A* **5**, 21242-21248 (2017).

12 Kim, J. H. *et al.* Overall photoelectrochemical water splitting using tandem cell under simulated sunlight. *ChemSusChem* **9**, 61-66 (2016).

13 Hayashi, T. *et al.* Powder-based $(CuGa_{1-y}In_y)_{1-x}Zn_{2x}S_2$ solid solution photocathode with a largely positive onset potential for solar water splitting. *Sustainable Energy & Fuels* **2**, 2016-2024 (2018).

14 Huang, D. *et al.* Over 1% efficient unbiased stable solar water splitting based on a sprayed Cu_2ZnSnS_4 photocathode protected by a HfO_2 photocorrosion-resistant film. *ACS Energy Lett.* **3**, 1875-1881 (2018).

15 Chen, M. *et al.* Spatial control of cocatalysts and elimination of interfacial defects towards efficient and robust CIGS photocathodes for solar water splitting. *Energy Environ. Sci.* **11**, 2025-2034 (2018).

16 Kobayashi, H. *et al.* Development of highly efficient $CuIn_{0.5}Ga_{0.5}Se_2$-based photocathode and application to overall solar driven water splitting. *Energy Environ Sci.* **11**, 3003-3009 (2018).

17 Kaneko, H., Minegishi, T., Kobayashi, H., Kuang, Y. & Domen, K. Suppression of poisoning of photocathode catalysts in photoelectrochemical cells for highly stable sunlight-driven overall water splitting. *J. Chem. Phys.* **150**, 041713 (2018).

18 Bornoz, P. *et al.* A Bismuth vanadate–cuprous oxide tandem cell for overall solar water splitting. *J. Phys. Chem. C* **118**, 16959-16966 (2014).

19 Pan, L. *et al.* Boosting the performance of Cu_2O photocathodes for unassisted solar water splitting devices. *Nat. Cat.* **1**, 412-420 (2018).

20 Kim, J. H. *et al.* All-bismuth-based oxide tandem cell for solar overall water splitting. *ACS Appl. Energy Mater.* **1**, 6694-6699 (2018).

21 Andrei, V. *et al.* Scalable triple cation mixed halide perovskite–$BiVO_4$ tandems for bias-free water splitting. *Adv. Energy Mater.* **8**, 1801403 (2018).

22 Zhou, Y. *et al.* Thin-film Sb_2Se_3 photovoltaics with oriented one-dimensional ribbons and benign grain boundaries. *Nat. Photon.* **9**, 409 (2015).

23 Wang, L. *et al.* Stable 6%-efficient Sb_2Se_3 solar cells with a ZnO buffer layer. *Nat. Energy* **2**, 17046 (2017).

24 Kumagai, H. *et al.* Efficient solar hydrogen production from neutral electrolytes using surface-modified Cu(In,Ga)Se_2 photocathodes. *J. Mater. Chem. A* **3**, 8300-8307, (2015).

25 C. A. Rodriguez et al., Design and cost considerations for practical solar-hydrogen generators, *Energy Environ. Sci.*, **7**, 3828-3835 (2014).

26 M. R. Shaner et al., A comparative technoeconomic analysis of renewable hydrogen production using solar energy, *Energy Environ. Sci.*, **9**, 2354-2371 (2016).

27 J. Kibsgaard & I. Chorkendorff, Considerations for the scaling-up of water splitting catalysts, *Nat. Energy.*, **4**, 430-433 (2019).

28 Kemppainen, E. *et al.* Scalability and feasibility of photoelectrochemical H_2 evolution: the ultimate limit of Pt nanoparticle as an HER catalyst. *Energy Environ. Sci.* **8**, 2991-2999 (2015).

29 Yang, W., Prabhakar, R. R., Tan, J., Tilley, S. D., Moon, J., Strategies for enhancing the photocurrent, photovoltage, and stability of photoelectrodes for photoelectrochemical water splitting. *Chem. Soc. Rev.*, **48**, 4979 (2019).

30 Tan, J. *et al.* Fullerene as a photoelectron transfer promoter enabling stable TiO_2-protected Sb_2Se_3 photocathodes for photo-electrochemical water splitting. *Adv. Energy Mater.* **9**, 1900179 (2019).

31 Laskowski, F. A., Nellist, M. R., Qiu, J. & Boettcher, S. W. Metal oxide/(oxy) hydroxide overlayers as hole collectors and oxygen-evolution catalysts on water-splitting photoanodes. *J. Am. Chem. Soc.* **141**, 1394-1405 (2018).

32 Ma, Y., Kafizas, A., Pendlebury, S. R., Le Formal, F. & Durrant, J. R. Photoinduced absorption spectroscopy of CoPi on $BiVO_4$: the function of CoPi during water oxidation. *Adv. Funct. Mater.* **26**, 4951-4960 (2016).

33 Toma, F. M. et al. Mechanistic insights into chemical and photochemical transformations of bismuth vanadate photoanodes. *Nat. comm.* **7**, 12012 (2016).

34 Lee, D. K. & Choi, K.-S. Enhancing long-term photostability of $BiVO_4$ photoanodes for solar water splitting by tuning electrolyte composition. *Nat. Energy* **3**, 53 (2018).
